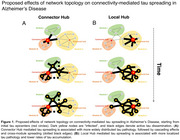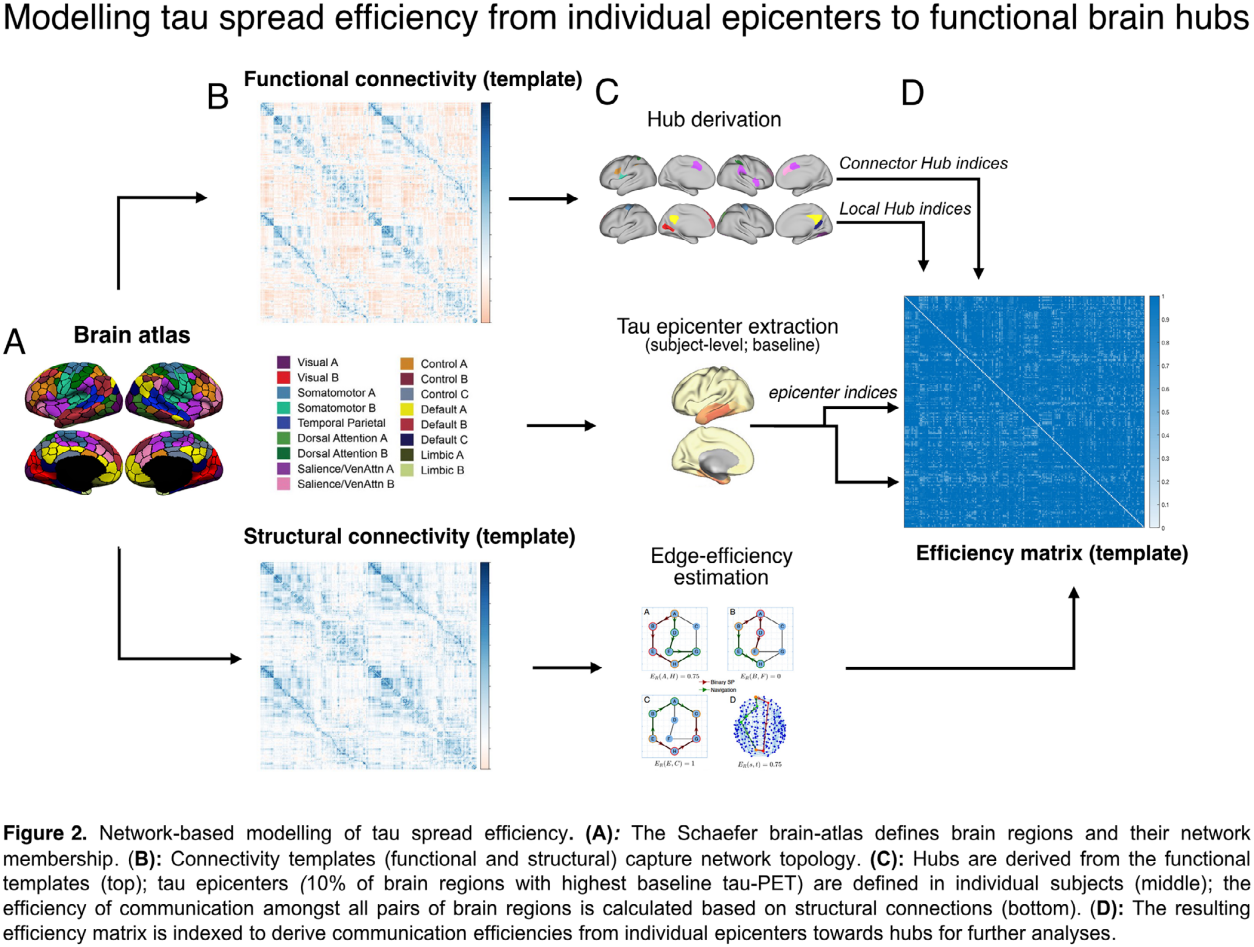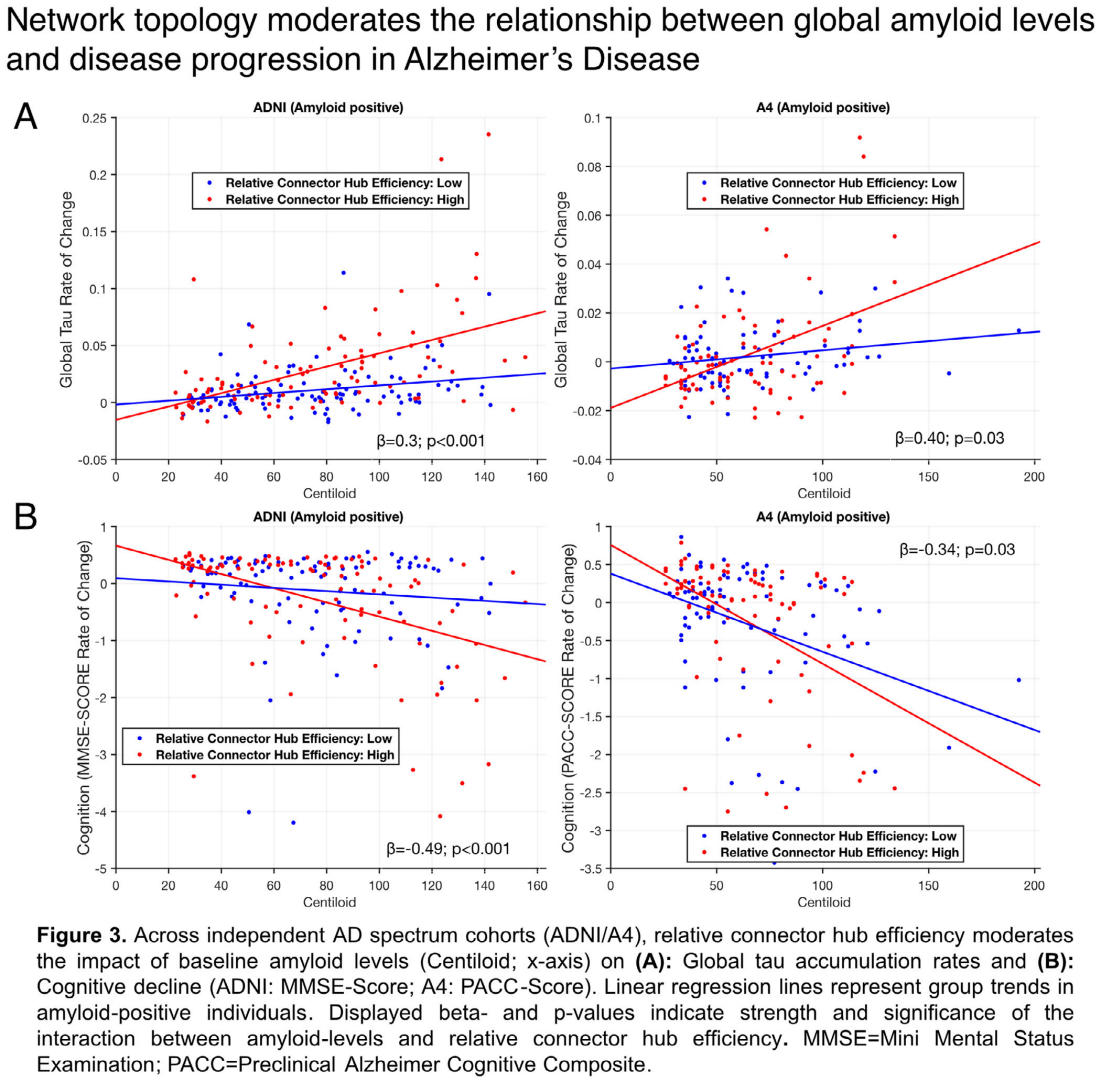# Connector Hubs Accelerate the Spread of Tau Pathology in Alzheimer's Disease

**DOI:** 10.1002/alz70862_109933

**Published:** 2025-12-23

**Authors:** Fabian Hirsch, Lukas Frontzkowski, Sebastian Roemer‐Cassiano, Amir Dehsarvi, Anna Steward, Anna Dewenter, Davina Biel, Madleen Klonowksi, Zeyu Zhu, Johannes Gnörich, Michael Schöll, Günter U Höglinger, Matthias Brendel, Nicolai Franzmeier

**Affiliations:** ^1^ Institute for Stroke and Dementia Research (ISD), University Hospital, LMU Munich, Munich, Bavaria Germany; ^2^ University Hospital, LMU Munich, Munich, Bavaria Germany; ^3^ Department of Nuclear Medicine, University Hospital, LMU Munich, Munich, Bavaria Germany; ^4^ Department of Neurology, University Hospital, LMU Munich, Munich, Bavaria Germany; ^5^ Max Planck School of Cognition, Leipzig, Sachsen Germany; ^6^ Institute for Stroke and Dementia Research (ISD), University Hospital, LMU Munich, Munich Germany; ^7^ Department of Nuclear Medicine, University Hospital, LMU Munich, Munich, Germany, Munich Germany; ^8^ Department of Psychiatry and Neurochemistry, Institute of Neuroscience and Physiology, The Sahlgrenska Academy, University of Gothenburg, Mölndal Sweden; ^9^ Wallenberg Centre for Molecular and Translational Medicine, Gothenburg Sweden; ^10^ Dementia Research Centre, UCL Queen Square Institute of Neurology, London UK; ^11^ Department of Neurology, Klinikum der Ludwig‐Maximilians Universität München, Munich Germany; ^12^ Munich Cluster for Systems Neurology (SyNergy), Munich, Bavaria Germany; ^13^ German Center for Neurodegenerative Diseases (DZNE), Munich Germany; ^14^ University of Gothenburg, The Sahlgrenska Academy, Institute of Neuroscience and Physiology, Psychiatry and Neurochemistry, Gothenburg Sweden; ^15^ Institute for Stroke and Dementia Research (ISD), LMU University Hospital, LMU, Munich, Bavaria Germany

## Abstract

**Background:**

Tau accumulation drives neurodegeneration and cognitive decline in Alzheimer’s Disease (AD) and preclinical research suggests that tau spreads transsynaptically across connected neurons. We translated tau spreading models to human neuroimaging data, showing that tau pathology spreads from circumscribed epicenters to connected regions in AD, following the architecture of functional brain networks. To further determine whether the topology of brain networks influences tau spreading dynamics, we investigated whether functional hubs (i.e. regions with strong inter‐regional connections) accelerate tau spread in AD. Specifically, we hypothesized that more efficient communication from tau epicenters towards hubs that cross‐link large‐scale brain networks (connector hubs) rather than hubs that interconnect neighboring regions (local hubs) accelerates amyloid‐related tau accumulation and cognitive decline (Figure 1).

**Method:**

Longitudinal tau/amyloid‐PET and cognitive data from two independent cohorts covering the AD spectrum (ADNI/A4 *n* = 325/220) were analyzed to examine amyloid‐driven spatiotemporal tau accumulation patterns and cognitive decline. Structural‐ and functional‐connectivity templates from healthy controls were used to model the connectional efficiency of subject‐level tau epicenters (i.e. 10% of brain regions with highest baseline tau‐PET) towards connector/local hubs (Figure 2). Using robust regression, we then tested whether more efficient communication of subject‐level tau epicenters to connector vs. local hubs accelerated global tau accumulation, cognitive decline, and tau dissemination across networks.

**Result:**

Supporting our hypotheses, we found that the effect of higher baseline amyloid‐PET on faster global tau‐PET increases was moderated by more efficient communication of tau epicenters towards connector relative to local hubs (ADNI/A4: *β* = 0.31/0.40, *p*<0.001/0.03), such that subjects with stronger epicenter communication to connector hubs showed an amplified effect of amyloid on global tau accumulation rates (Figure 3A). The same interaction models also predicted faster cognitive decline (ADNI/A4: *β* = ‐0.49/‐0.34, *p*<0.001/0.04, Figure 3B), and larger extents of tau dissemination across functional networks (ADNI/A4: *β* = 0.6/0.36, *p*<0.001/0.04). All *p*‐values were FDR‐corrected.

**Conclusion:**

Brain network topology shapes spatiotemporal tau accumulation rates and cognitive trajectories in AD. Specifically, stronger communication of tau epicenters with connector hubs that are characterized by widespread cross‐network connections amplifies amyloid‐related tau accumulation. This suggests that brain network architecture has a profound modulating impact on tau aggregation and disease progression in AD.